# Effect of the number of abutments on biomechanics of Branemark prosthesis with straight and tilted distal implants

**DOI:** 10.1590/S1678-77572010000200013

**Published:** 2010

**Authors:** Marcos Michelon NACONECY, Tomás GEREMIA, André CERVIERI, Eduardo Rolim TEIXEIRA, Rosemary Sadami SHINKAI

**Affiliations:** 1 DDS, MSc, PhD, Research Fellow, Graduate Program in Dentistry, Pontifical Catholic University of Rio Grande do Sul, Porto Alegre, RS, Brazil.; 2 DDS, MSc, Assistant Professor, Department of Prosthodontics, Pontifical Catholic University of Rio Grande do Sul, Porto Alegre, RS, Brazil.; 3 Eng., MSc, Ph.D, Associate Professor, Department of Engineering, Lutheran University of Brazil, Canoas, RS, Brazil.; 4 DDS, MSc, PhD, Associate Professor, Department of Prosthodontics, Pontifical Catholic University of Rio Grande do Sul, Porto Alegre, RS, Brazil.

**Keywords:** Biomechanics, Implant-supported prostheses, Number of abutments, Tilted implants, Strain gauges

## Abstract

**Objective:**

This study aimed to evaluate the bending moments, and compressive and tensile forces in implant-supported prostheses with three, four or five abutments.

**Material and Methods:**

Ten Pd-Ag frameworks were tested over two master models with: 1) parallel vertical implants, and 2) tilted distal implants. Strain gauges were fixed on the abutments of each master model to measure the deformation when a static load of 50 N was applied on the cantilever (15 mm). The deformation values were measured when the metallic frameworks were tested over three, four or five abutments, and transformed into force and bending moment values. Data were analyzed by ANOVA and Tukey’s test for multiple comparisons at 5% level of significance.

**Results:**

Abutment #1 (adjacent to the cantilever) had the highest values of force and sagittal bending moment for all tests with three, four or five abutments. Independently from the number of abutments, axial force in abutment #1 was higher in the vertical model than in the tilted model. Total moment was higher with three abutments than with four or five abutments. Independently from the inclination of implants, the mean force with four or five abutments was lower than that with three abutments.

**Conclusion:**

The results suggest that in the set-ups with four or five abutments tilted distal implants reduced axial force and did not increase bending moments.

## INTRODUCTION

Studies on the biomechanics of different designs of implant-supported prostheses may help maximizing the clinical outcomes of implant treatment. Five to six implants and distal cantilever were traditionally indicated to rehabilitate the edentulous mandible and maxilla by means of fixed implant-supported prosthesis^6^. More recently, clinical reports have shown shortand medium-term success using less implants combined or not with inclination of distal implants^4,7,19-21^. Nevertheless, it still is controversial how many implants would be necessary to support a fixed implant-supported prosthesis with greater predictability. Also, no experimental data are currently available showing biomechanical gain with combination of implant inclination and reduction of the number of implants.

The technique of implant inclination was introduced for selected cases of multiple implants in the edentulous maxilla and mandible. In the mandible, the procedure can be used when the mental foramen is positioned low in relation to the alveolar ridge to reduce the cantilever extension and increase the polygonal area of prosthesis support^19,23^. In maxilla with bone atrophy and presence of large sinuses, longer inclined implants can be placed in areas of high bone density, with emergency at the first molar region. Without using tilted implants, these regions would receive shorter implants or would need maxillary sinus floor augmentation or bone grafting, increasing the treatment complexity, time, and costs^1,5,8^.

Experimental strain levels transmitted to tilted implants and surrounding bone and the deformation of prosthetic components still are unclear. The axial and non-axial forces generated during oral function may result from sommatory, synergistic or antagonic effects of implant inclination and number and distribution of implants in the arch. Strain gauges studies^12,13,15^ as well as mathematical^23^, photoelastic^25^ and finite element models^2,3^ have been used to explain the biomechanical behavior of implant-supported prostheses simulating the variation of number or inclination of implants, yet no experimental study has evaluated the combined effect of these variables. Therefore, this study used strain gauges to assess the effect of the number of abutments and inclination of distal implants on axial forces and bending moments in implantsupported prostheses.

## MATERIAL AND METHODS

Two trapezoid epoxy resin bases were used to fabricate the master model with vertical (straight) implants and the master model with posterior tilted implants. The arch (curve of 134.30º and radium of 17.61 mm) of a mandible model (ETH 0301-10 Nobel Biocare, Gothenburg, Sweden) was transposed to the epoxy bases for the perforation of the implant sites. For the model with vertical implants, the central implant site was marked at the sagittal line; five perforations (4 mm-diameter, 17 mm-length) were made parallel and 1 cm apart from each other. For the model with tilted implants, the three central perforations were made vertical, and the two posterior perforations were tilted using an index with a 27-degree inclination plane. Ten 4.0 x 15mm screw-type implants (OSS 415, 3i Implant Innovations, Palm Beach Gardens, FL, USA) were fixed into the perforations with fluid epoxy resin. After 12 h, ten 7-mm standard abutments (AB700, 3i Implant Innovations, Palm Beach Gardens, FL, USA) were attached with 20 Ncm torque.

**Figure 1:**
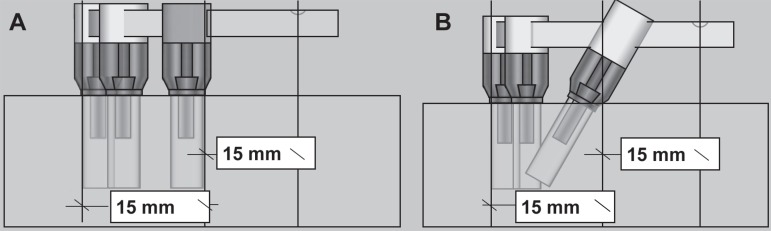
Scheme of the lateral view of the assembly with vertical implants (A) and with tilted distal implants (B). For both models, the sagittal distance between the most anterior and posterior points of the implant platform was 15 mm. The loading site on the cantilever was 15 mm from the emergency of the distal implant

Over each master model, five bars (rectangular section, 3-mm width, 4-mm height, and 20-mm of cantilever at the left side) were waxed up 1 mm above the epoxy base. The cantilever was placed on the left side beginning at the emergency point of the posterior implant. The wax patterns were sectioned into five segments and cast in a Pd-Ag alloy (Porson 4, Degussa, Dusseldorf, Germany) according to standard procedures. After finishing, the bar segments were laser-welded (EV LASER 900, Bergamo, Italy). The dimensions of the metallic bars were verified using digital calipers, and passivity of fit of the welded framework was checked by tightening one screw at time. The loading point on the cantilever was standardized at a 15-mm distance from the posterior emergency of the distal implant (Figure 1). With a milling machine, a concave notch was made with half-depth of a round tungsten bur of 2mm diameter. This notch matched the load applicator tip (2-mm diameter) of a customized mechanical device used to deliver the 50 N static load during the tests.

Three strain gauges (KFG02-120C1-11N15C2, Kyowa Electronic Instruments Co Ltd, Tokyo, Japan) were attached to the abutment surface, 120º apart, in the following geometric position: one anterior, one posterior to the right, and one posterior to the left. The 0.2-mm measuring grid was placed 1 mm above the implant platform in parallel with the axes of the cylinders. One strain gauge formed one channel for reading deformation (1/4 of a Wheatstone bridge). Therefore, 15 reading channels were built for each master-model (three per abutment). Each strain gauge was connected to two cables leading the signals to a 15-channel strain gauge conditioner (MGC Plus, HBM Inc, Berlin, Germany). The analogical signal of electric resistance variation was converted into a digital signal via a 12-byte resolution converter (MGC Plus, HBM Inc, Berlin, Germany). These signals were software-processed (MGC Plus, HBM Inc, Berlin, Germany), and channel signals originally measured in millivolts were converted into microstrain units (µm/m).

**Figure 2:**
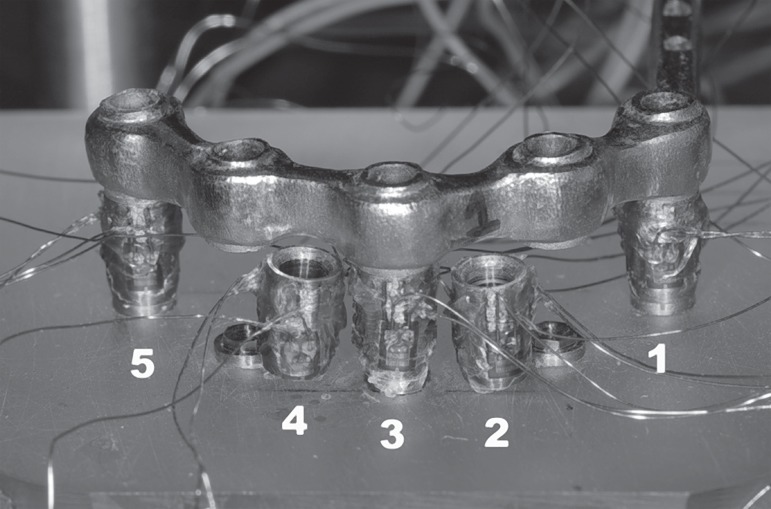
Measuring set-up for the three-implant configuration with the model with vertical implants. Abutments #2 and #4 were removed to perform the loading test of the framework supported by three abutments. Each abutment had three strain gauges attached 120º apart

Each framework was screwed (GS300; 3i Implant Innovations, Palm Beach Gardens, FL, USA) onto the respective master model with a 10 Ncm torque (DEC 600-1 Ossecare Drilling Equipment, and DIA 189-0, Nobel Biocare AB, Gothenburg, Sweden). The abutments were numbered clockwise (#1 to #5; abutment #1 was adjacent to the cantilever), and the tightening sequence was 2, 4, 3, 1, and 5^18^. A new set of screws was used for each framework to avoid screw fatigue. After the strain gauges were calibrated to zero, a 50 N static load was applied on the cantilever generating a graph of deformation. The point of signal stabilization was selected, and the deformation values were extracted. This test procedure was performed for all five frameworks supported by five abutments, then repeated with the frameworks supported by four or three implants/abutments. For the four-implant configuration, the central abutment (abutment #3) was removed. For the threeimplant configuration, abutments #2 and #4 were removed (Figure 2).

The readings of the strain gauges (deformation in microstrain unit) were transformed into normal axial force and bending moments around the Xand Y-axis using the calibration method and equations described by Duyck, et al.^12,13^ All abutments were individually calibrated with a 50 N static load to the implant/abutment axis. This calibration was performed by loading a customcast disc fixed to each abutment in five standardized positions so that the axial force (in relation to the abutment axis) and bending moments (sagittal and lateral) were computed separately. For axial force, a positive signal was conventionally adopted for compressive force and a negative signal for tensile force; all calculations were performed using the absolute values. Data were analyzed by Analysis of Variance for random blocks design using the Proc Mixed tool of the software SAS 9.1 - Type 3 Tests of Fixed Effects, followed by pairwise comparisons Tukey’s tests. A significance level of 0.05 was used for all tests.

## RESULTS

**Figure 3:**
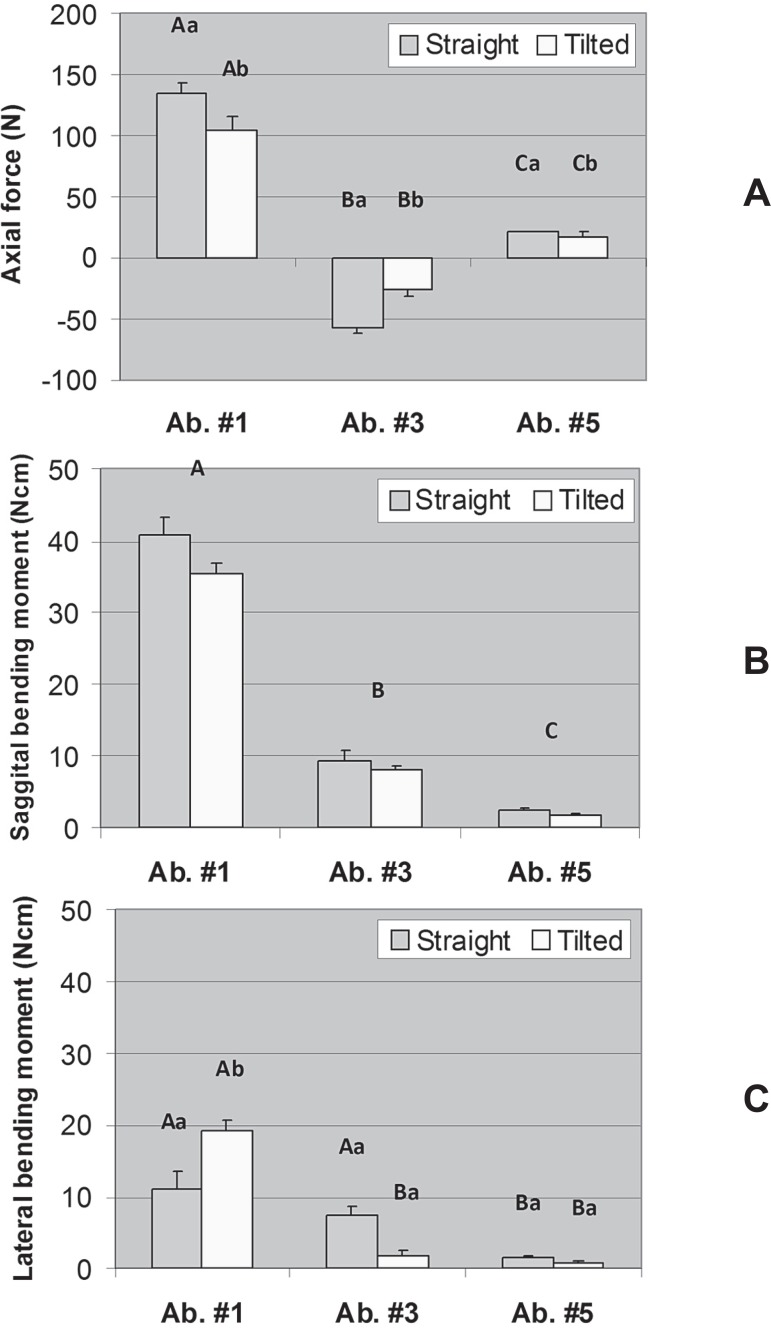
Axial force (A), sagittal bending moment (B), and lateral bending moment (C) in each abutment for the model with three abutments (#1, #3, and #5). Error bars are standard error of the mean. Distinct letters (uppercase letters for the factor “Model” and lowercase letters for the factor “Abutment”) indicate that the means are significantly different (α=0.05). For axial force no significant interaction was found between Model and Abutment (*P*=0.070) but both main effects were significant (Model: *P*=0.006; Abutment: *P*<0.001). For sagittal bending moment, only the factor Abutment was significant (*P*<0.001). For lateral bending moment, there was significant interaction between Model and Abutment (P<0.001)

**Figure 4:**
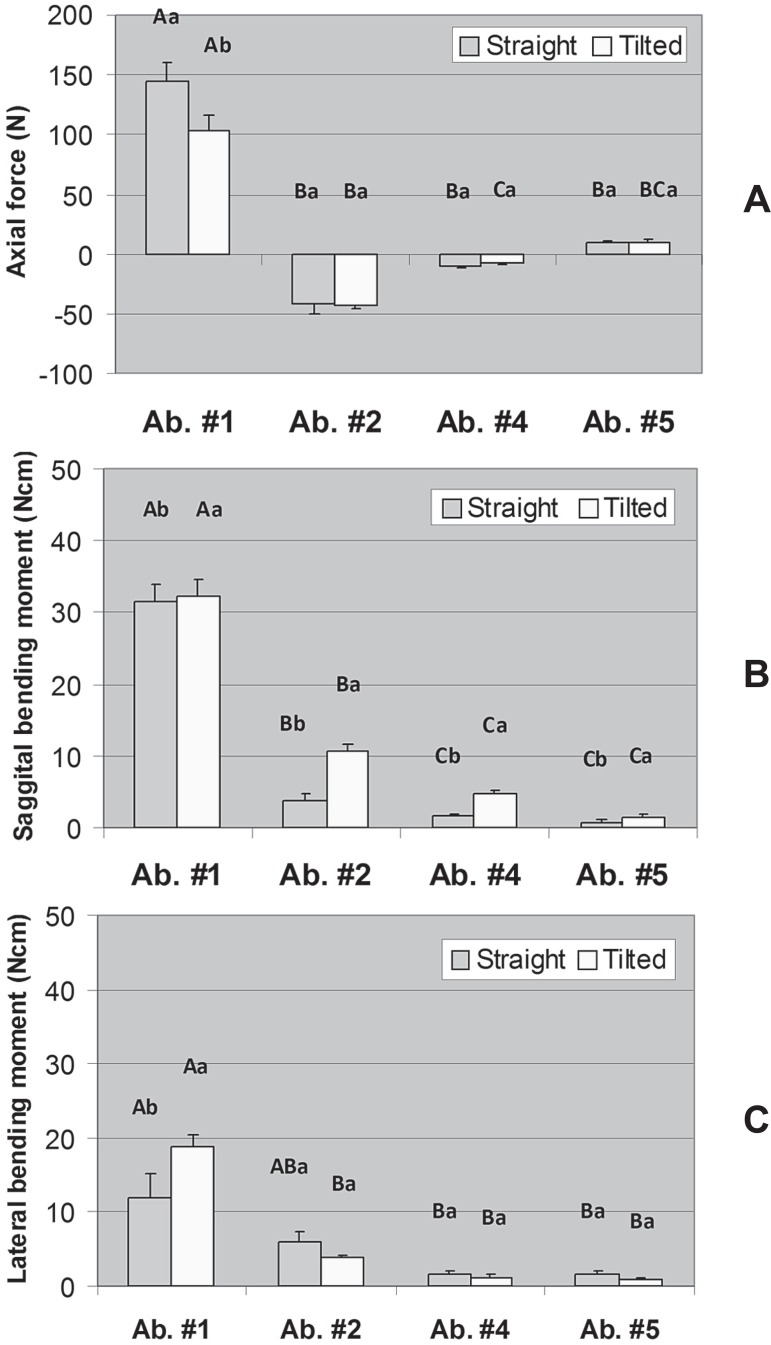
Axial force (A), sagittal bending moment (B), and lateral bending moment (C) in each abutment for the model with four abutments (#1, #2, #4, and #5). Error bars are standard error of the mean. Distinct letters (uppercase letters for the factor “Model” and lowercase letters for the factor “Abutment”) indicate that the means are significantly different (α=0.05). For axial force, there was significant interaction between Model and Abutment (*P*=0.029). For sagittal bending moment, there was no interaction between Model and Abutment (*P*=0.052) but both main effects were significant (Model: *P*=0.018; Abutment: *P*<0.001). Regarding lateral bending moment, there was a significant interaction between Model and Abutment (*P*=0.018)

**Figure 5:**
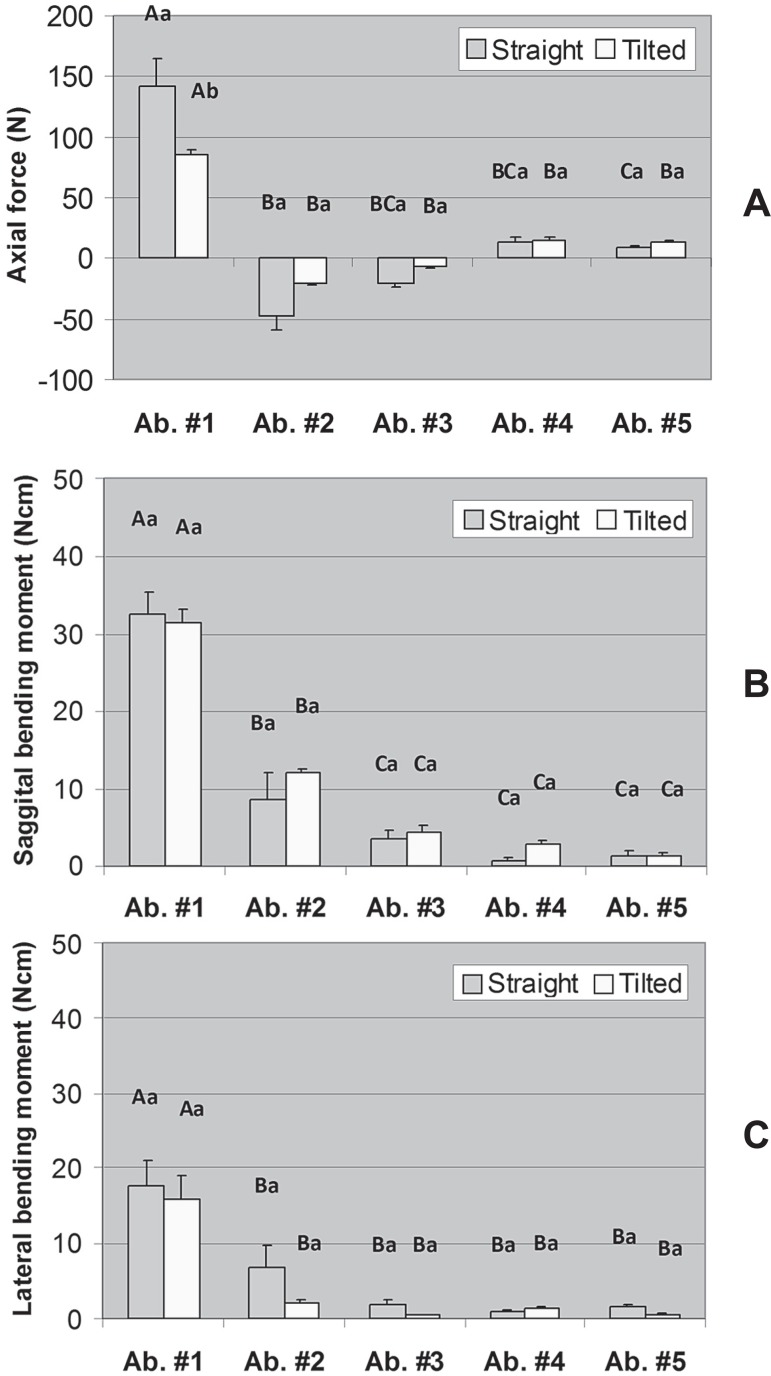
Axial force (A), sagittal bending moment (B), and lateral bending moment (C) in each abutment for the model with five abutments (#1, #2, #3, #4, and #5). Error bars are standard error of the mean. Distinct letters (uppercase letters for the factor “Model” and lowercase letters for the factor “Abutment”) indicate that the means are significantly different (α=0.05). For axial force there was significant interaction between Model and Abutment (*P*<0.001). For sagittal and lateral bending moments, only the factor Abutment was significant (*P*<0.001)

Figures 3, 4, and 5 display the mean axial force, sagittal bending moment, and lateral bending moment in each abutment for the models with three, four, and five abutments, respectively. For the model with three abutments, both main effects were significant for axial force (Model: P=0.006; Abutment: P<0.001), only the factor Abutment was significant for sagittal bending moment (P<0.001), and there was a significant interaction between Model and Abutment for lateral bending moment (P<0.001). For the model with four abutments, a significant interaction between Model and Abutment was found for axial force (P=0.029) and lateral bending moment (P=0.018); for sagittal bending moment, both main effects were significant (Model: P=0.018; Abutment: P<0.001). Regarding the model with five abutments there was a significant interaction between Model and Abutment for axial force (P<0.001), but only the factor Abutment was significant for sagittal and lateral bending moments (P<0.001).

**Figure 6:**
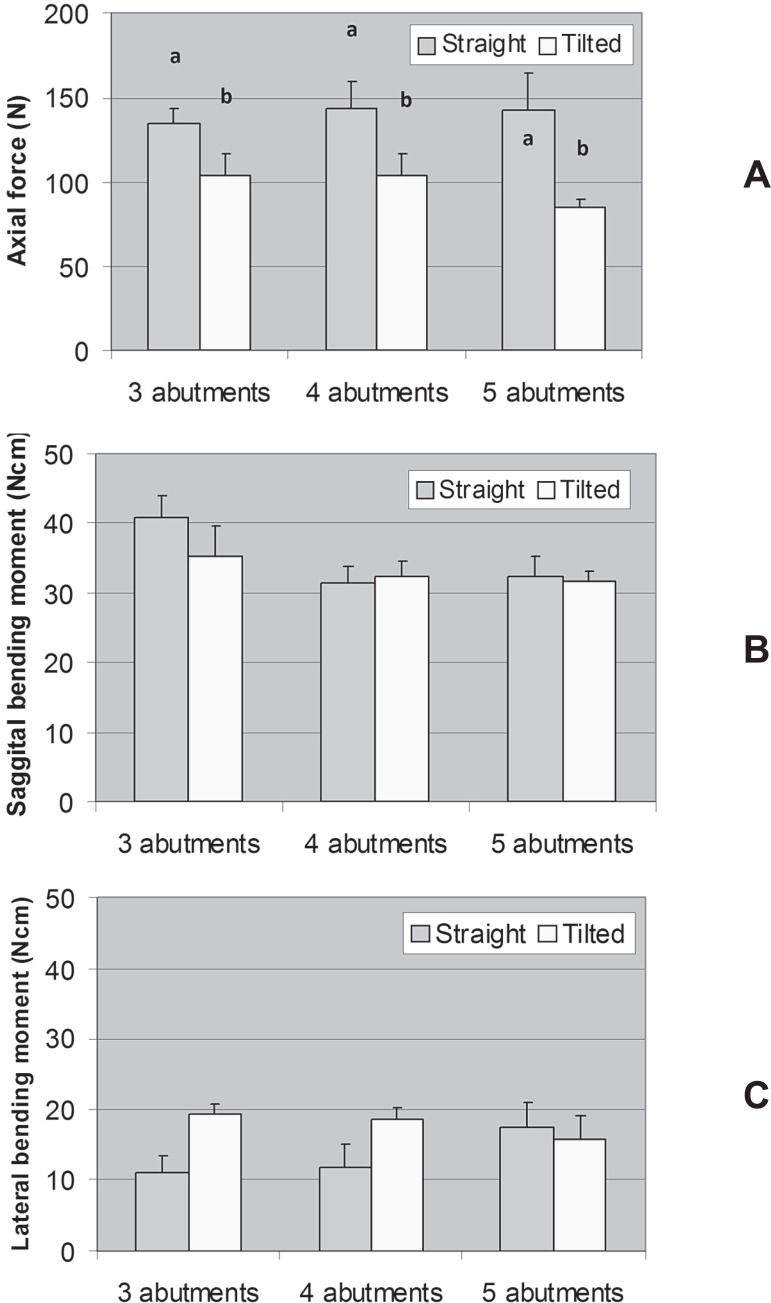
Axial force (A), sagittal bending moment (B), and lateral bending moment (C) in the abutment adjacent to the cantilever under loading (abutment #1). Error bars are standard error of the mean. For force values, a positive signal means compressive force and a negative signal means tensile force. Distinct letters above the bars of axial force means indicate that the means are significantly different (α=0.05). Sagittal and lateral bending moment means were not statistically different

Figure 6 shows the results for abutment #1. For axial force, interaction between Model and Number of abutments (P=0.623) and the main factor Number of abutments (P=0.759) were not significant. Only the factor Model was significant (P=0.001): the straight model had higher mean force than the tilted model. Sagittal and lateral bending moment means were not statistically different.

## DISCUSSION

This study showed that four or five abutments provided better distribution of forces and bending moments compared with the configuration with three abutments. Overall, the inclination of distal implants reduced the axial force and bending moments independently from the number of abutments. In relation to the direction of axial forces, compressive forces were measured in the distal abutments and tensile forces in the most anterior abutments in the arch in accordance with the “hinging effect” proposed by Duyck, et al.^13^.

The magnitude of forces measured with four or five abutments was similar and lower than that with three abutments. Davis, Zarb and Chao^11^ also observed the highest deformation of frameworks supported by only two abutments, but the distribution of axial forces and bending moments were similar when four or five abutments were used. Conversely, Duyck, et al.^13^ found *in vivo* lower forces with five implants in comparison with the arrangements with four or three implants. Some of these discrepancies may be explained by the difference of interimplant distances that alter the geometric distribution of abutments in the arch and the length of framework segments. The magnitude of resulting forces also depends on the deformation of the entire system, which may be influenced by the elastic deformation of the framework^17^ and by the material used to fabricate the prosthetic screws, which influence preload and torque values^24^.

In the present study, the values of bending moment were lower than those of axial force independently from the number of abutments, but higher bending moments were usually observed with fewer abutments. Conversely, Glantz, et al.^15^ recorded higher values of bending moments than axial forces during maximal biting *in vivo*, which may be explained by the distribution of occlusal contacts and resulting nonaxial forces.

The results observed for the framework supported by three abutments suggest that, although the central abutment provided a longer resistance arm (15 mm) than the configuration with four abutments (11.35 mm), the quadrilateral polygon resulted in better distribution of forces. This was more evident for the non-axial forces represented by the sagittal and lateral bending moments. Other biomechanical studies using an analytical mathematical model^23^ and finite elements analysis^2^ demonstrated that a spread-out arrangement of implants in the arch is more significant than the number of implants *per se* for the distribution of masticatory forces.

The tilted implants/abutments were calibrated with a 50 N load axial to the implant/abutment axis and non-perpendicular to the metallic bar. Therefore, this study measured the axial force in relation to the implant/abutment axis to evaluate the effect of implant inclination. The inclination of distal implants reduced the axial forces in all abutments and also in abutment #1 when four or five abutments were used. This inclination allowed simultaneous reduction of the cantilever length at the connection abutmentframework and increase of the prosthesis support area. Using a mathematical model, Skalak^23^ predicted that the inclination of 30 degrees of the distal implant considerably reduces the forces in a configuration with three implants. In mandibular prostheses, the fulcrum of rotation for the distal implant depends on the anatomical position of the mental foramen in relation to the alveolar ridge. The more apical the foramen, the more apical the fulcrum. In this experiment, the distal implants were inclined having the implant platform as the fulcrum of rotation, and the implant was not displaced distally any further. However, considering the connection frameworkabutment, the use of 7-mm abutments reduced the cantilever length from 15 to 12.16 mm. This reduction of the cantilever length is inherent to the implant tilting. This *in vitro* study aimed to assess the effect of tilting the distal implant per se on the axial forces and bending moments of the system as the tilted implant platforms did not emerge more distally in relation to the vertical model cast. This was designed to allow the evaluation of the isolated variable inclination without the combined effect of further distalizing the implant platform. The results in abutment #1 were of particular interest and showed that not only the axial force is lower on the tilted abutment but also the sagittal bending moment did not increase. Because the present study did not vary the position of the distal platform, this finding can be attributed to the variable implant inclination. If it had chosen to position the tilted implant more distally, it would not be possible to isolate the effect of implant inclination.

The main strength of the paper is that this is the first experimental work to prove that tilting the distal implants may offer a biomechanical advantage over vertical implants when cantilever is needed. Previous works used an analytical method or finite element analysis, which simulate experimental conditions to some extent and require several simplifications of the geometric models and mathematical approach. However, this study has some limitations because the experiment simulated a specific design of a fixed implant-supported prosthesis for the edentulous mandible. Previous studies^10,16,22^ have highlighted that the load transference from implants to bone depends on the type and place of loading, boneimplant interface, implant geometry and surface, framework alloy, density of cancellous bone, and abutment length. The literature also reports variation of deformation, forces and/or bending moments in abutments and implants due to implant brand^9^, framework alloy^22^, cantilever extension^17^, and occlusal contacts^14^.

Another limitation of the present study is inherent to the strain gauge size and placement, which do not allow measurement of the forces and bending moments directly in implants and bone interface. Furthermore, the absolute values of forces and bending moments are valid only for the present study set-up. The absolute values of forces and bending moments in abutments cannot be directly generalized to implants because of the joints and gaps among prosthetic components, screws, and implants. However, theoretically one can expect having similar vectors and biomechanical behavior in tilted implants and abutments as long as the abutment and the implant are aligned in the same longitudinal axis.

## CONCLUSIONS

The present results suggest that tilted distal implants reduce axial force and do not increase bending moments when four or five abutments are used. Further controlled clinical studies are necessary to evaluate the combined effect of the number of abutments and inclination of distal implants on short- and long-term success in the daily practice.
